# Supplementary material for: TNF-α-Mediated Endothelial Cell Apoptosis Is Rescued by Hydrogen Sulfide
Source: Antioxidants (Basel). 2023 Mar 16;12(3):734. doi: 10.3390/antiox12030734 (PMC10045727; doi:10.3390/antiox12030734)
Supplement: Supplementary file 1 [file antioxidants-12-00734-s001.zip › antioxidants-2253842-supplementary.pdf]

Supplementary Figures

Table S1. List of forward and reverse primers sequences used in RT-qPCR analysis.

Target Gene	Forward 5'→ 3'	Reverse 5'→ 3'
**EEF2**	TGAACAAGATGGACCGCG	GGATCGATCATGATGTTGCC
**Trx1**	GTGAAGCAGATCGAGAGCAAG	CGTGGCTGAGAAGTCAACTAC
**DRP1**	AAGCTGCTGCCATAGTCCTC	ACCACAGCCATGTCAGTGTC
**MNF1**	GTCGCAAACTCTGAATCAACAC	ATCTTTCCATGTGCTGTCTGC
**SOD2**	TAGGGCTGAGGTTTGTCCAG	GGAGAAGTACCAGGAGGCGT

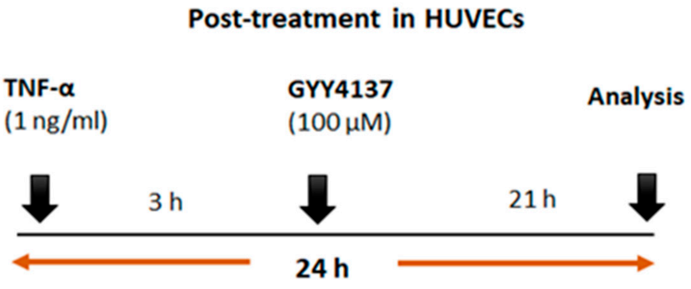

Figure S1. Scheme of the experimental schedule for HUVECs treated with TNF-α and GYY4137 (h=hours). Cells were treated with TNF-α (1 ng/ml, 3h) or vehicle (medium, untreated cells) followed by post-treatment with GYY4137 (100 μM, 21 h).

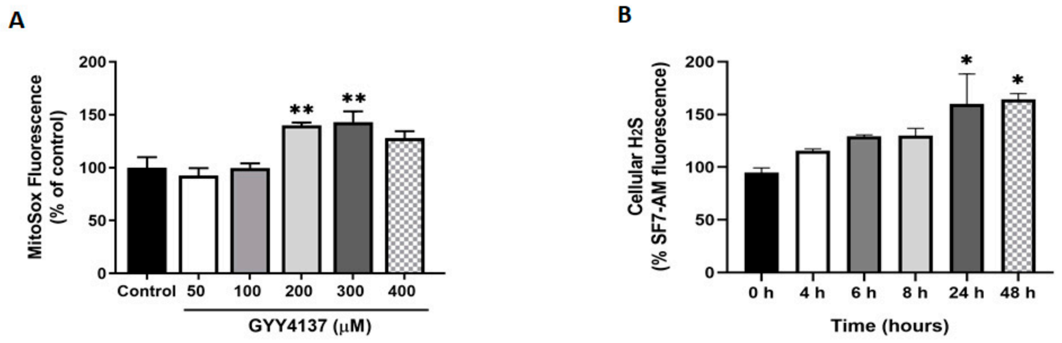

Figure S2. Determination of non-toxic concentration of GYY4137 and effect of H_2S content in HUVECs. (A) Quantification of superoxide formation in dose-dependent GYY4137 treatment in HUVECs by MitoSox staining. **(B)** Intracellular H_2S content in a time-dependent GYY4137 (100 μM) treatment in HUVECs. Statistic significant differences were calculated using the one-way ANOVA followed by Dunnett's comparison test. Data are shown as means ± SD; *$p<0.05$ and **$p<0.01$ vs. control, (n=3).

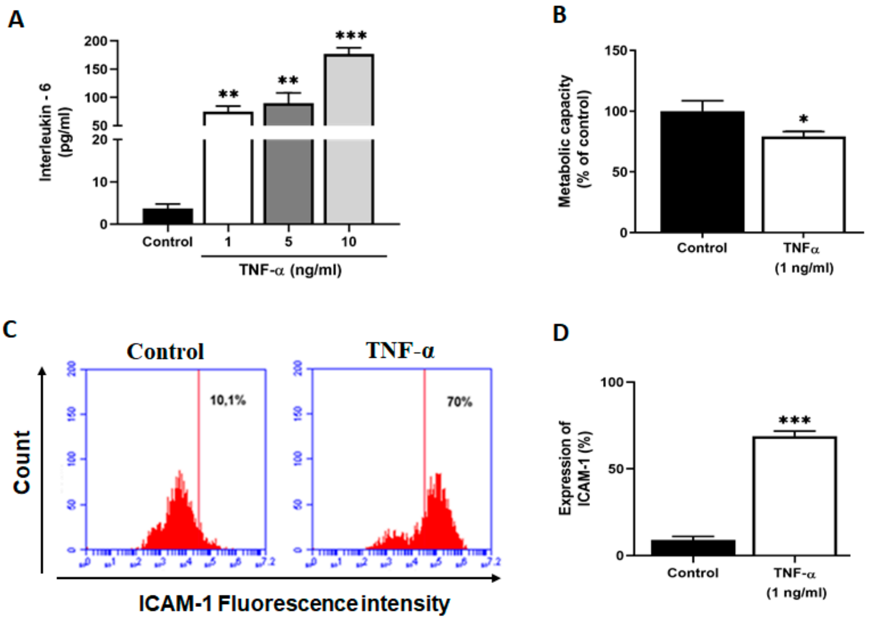

Figure S3. Activation of endothelial cells by TNF-α treatment. (A) HUVECs were treated with dose-dependent TNF-α for 3 h before supernatant was collected. Secretion of interleukin 6 (IL-6) was analysed by ELISA. **(B)** Metabolic activity of viable cells in the presence of TNF-α treatment by CellTiter Blue assay. **(C,D)** Intercellular cell adhesion molecule (ICAM-1) expression was analysed by cell stained with CD54 antibody (APC) and ICAM-1 expression in cells with or without TNF-α (1 ng/ml, 3h) was analysed by flow cytometry. Statistic significant differences were calculated using the Student *t*-test and one-way ANOVA followed by Dunnett's comparison test. Data are shown as means ± SD; *p<0.05,**p<0.01 and ***p<0.001 vs. control, (n=3).

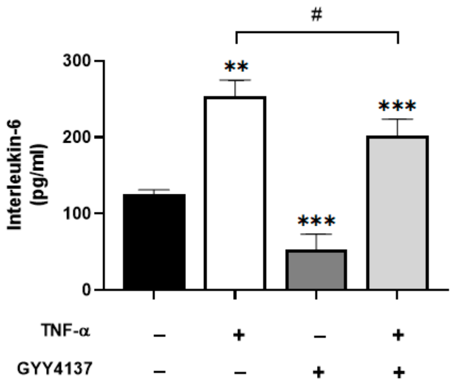

Figure S4. Effects of GYY4137 on IL-6 content in TNF-α-treated HUVECs. Endothelial cells were treated with TNF-α (1 ng/ml, 3h) followed by post-treatment with GYY4137 (100 μM, 21h). Supernatant was collected and IL-6 secretion was analysed by ELISA assay. Statistic significant differences were calculated using one-way ANOVA for Tukey's comparison test. Data are shown as means ± SD; **p<0.01 and ***p<0.001 vs. control, (n=3).

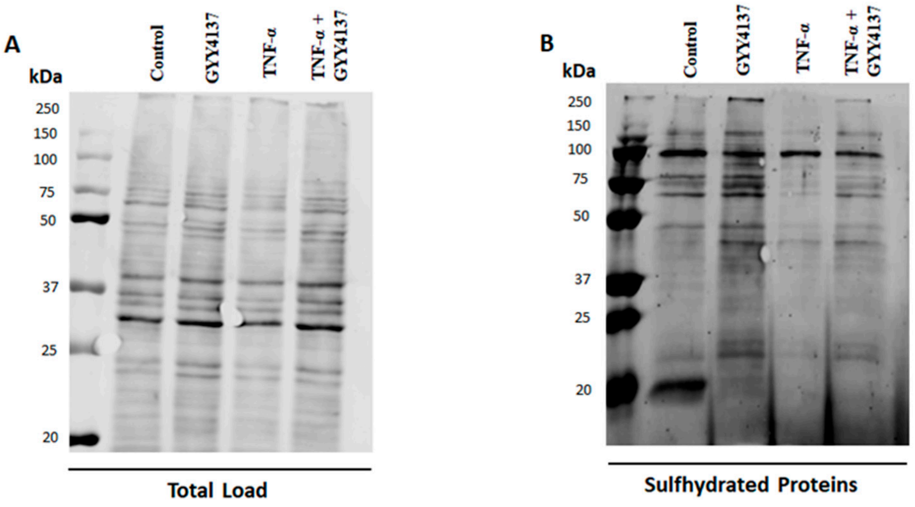

Figure S5. GYY4137 post-treatment increases sulfhydration of proteins. **(A)** detection of total proteins and **(B)** total S-sulfhydrated proteins after Biotin switch assay using Revert™ 700 Total protein stain kit.

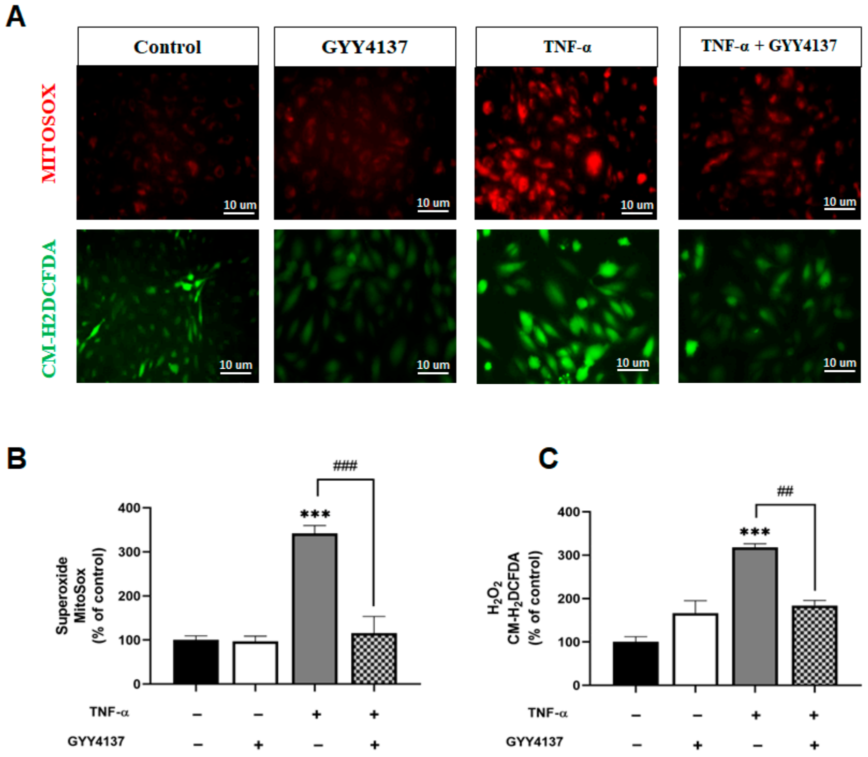

Figure S6. Effects of GYY4137 on oxidants in TNF-*α*-treated HUVECs. **(A)** Representative images of HUVECs stained with MitoSox probe (red) and CM-H2DCFDA probe (green). Scale bar 10 μm. **(B)** Quantification of superoxide and **(C)** H_2O_2 levels in endothelial cells. P-values were calculated using one-way ANOVA followed by Tukey's comparison test. Data are shown as means ± SD; ***p<0.0001 vs. control. ##p<0.01 and ###p<0.0001 vs. TNF-*α*-treated groups only (n=4-7).
